# Greater insulin sensitivity in calorie restricted rats occurs with unaltered circulating levels of several important myokines and cytokines

**DOI:** 10.1186/1743-7075-9-90

**Published:** 2012-10-15

**Authors:** Naveen Sharma, Carlos M Castorena, Gregory D Cartee

**Affiliations:** 1 Muscle Biology Laboratory, School of Kinesiology, University of Michigan, Ann Arbor, MI, USA; 2 Department of Molecular and Integrative Physiology, University of Michigan, Ann Arbor, MI, USA; 3 Institute of Gerontology, University of Michigan, Ann Arbor, MI, USA; 4 University of Michigan, School of Kinesiology, Room CCRB 2200, 401 Washtenaw Avenue, Ann Arbor, MI, 48109-2214, USA

**Keywords:** Myonectin, Myostatin, FGF21, Irisin, Insulin resistance, Dietary restriction, Adiponectin, FNDC5

## Abstract

Calorie restriction (CR; ~60% of ad libitum, AL intake) has been associated with substantial alterations in body composition and insulin sensitivity. Recently, several proteins that are secreted by nontraditional endocrine tissues, including skeletal muscle and other tissues, have been discovered to modulate energy metabolism, body composition, and insulin sensitivity. The aim of this study was to characterize the influence of CR by rats on plasma levels of six of these newly recognized metabolic hormones (BDNF, FGF21, IL-1β, myonectin, myostatin, and irisin). Body composition of 9-month old male Fischer-344/Brown Norway rats (AL and CR groups) was determined by nuclear magnetic resonance. Blood sampled from the carotid artery of unanesthetized rats was used to measure concentrations of glucose and plasma proteins. As expected, CR versus AL rats had significantly altered body composition (reduced percent fat mass, increased percent lean mass) and significantly improved insulin sensitivity (based on the homeostasis model assessment-estimated insulin resistance index). Also consistent with previous reports, CR compared to AL rats had significantly greater plasma levels of adiponectin and corticosterone. However, there were no significant diet-related differences in plasma levels of BDNF, FGF21, IL-1β, myonectin, myostatin, or irisin. In conclusion, these results indicate that alterations in plasma concentration of these six secreted proteins are not essential for the CR-related improvement in insulin sensitivity in rats.

## Findings

Moderate calorie restriction (CR; ~60% of ad libitum, AL, food consumption) has well-known effects on body composition, glucose homeostasis, plasma insulin concentration, insulin sensitivity, and other aspects of metabolic health in many species, including rats [1-3], mice [4,5], non-human primates [6], and humans [7]. Several circulating proteins have been recently discovered and found to modulate energy metabolism and insulin sensitivity. It seems possible that the healthful metabolic benefits of CR may be, at least in part, related to alterations in the plasma levels of some of these proteins. For example, the plasma concentration of adiponectin, an insulin-sensitizing adipokine, has been reported to be increased with CR in rats [1], mice [8] and humans [9].

We were especially interested in CR effects on plasma proteins that are secreted into the circulation by skeletal muscle (i.e., myokines) because insulin signaling in this tissue is highly responsive to CR [10]. CR induces enhanced insulin sensitivity in skeletal muscle [11,12] and skeletal muscle accounts for the largest portion of insulin-stimulated glucose disposal [13]. Myonectin (also known as C1q/TNF-related protein 15; CTRP15) [14], and irisin (recognized as a product of proteolytic cleavage of FNDC5) [15] are newly discovered myokines with metabolic functions. Circulating myonectin concentration in normal mice was reported to be responsive to fasting and refeeding, and treating these mice with recombinant myonectin lowered circulating non-esterified fatty acids levels [14]. Irisin was reported to induce a program of brown fat-like development in white adipose cells and to oppose high fat diet-induced obesity and insulin resistance in mice [15]. Myostatin is a myokine that is best known for its role in regulating skeletal muscle mass [16], but it can also influence metabolism and insulin sensitivity [17]. To the best of our knowledge, earlier studies have not assessed CR effects on any of these three myokines. A number of other plasma proteins that have been implicated as potential regulators of body composition and/or energy metabolism are expressed and apparently secreted by skeletal muscle and various other tissues. Several of these proteins that have not been previously assessed in the context of CR include brain-derived neurotrophic factor (BDNF) [18], fibroblast growth factor 21 (FGF21) [19], and interleukin-1β (IL-1β) [20].

The goal of the current study was to characterize the effects of moderate CR on plasma levels of myonectin, myostatin, irisin, BDNF, FGF21, and IL-1β in 9 month-old rats. These proteins were selected for study because they are newly discovered plasma myokines and/or cytokines that were recently recognized to have metabolic functions, and they have not been previously studied with CR. Accordingly, it seemed possible that they might play a role in the CR phenotype (e.g., increased insulin sensitivity). We also assessed other parameters that have been previously documented to be responsive to CR-induced changes (body composition, homeostasis model assessment-estimated insulin resistance [HOMA-IR] index, and plasma concentrations of insulin, C-peptide, adiponectin and corticosterone) [1,10,21-24]. We hypothesized that CR-related changes in these parameters would be accompanied by altered plasma values of one or more of these six proteins that have been recently linked to modulation of metabolism and insulin action.

Procedures for animal care were approved by the University of Michigan Committee on Use and Care of Animals. Male Fischer-344 x Brown Norway, F1 generation rats were obtained at 3 months of age from Harlan (Indianapolis, IN). Rats were housed individually in shoebox cages and maintained on a 12:12 h light–dark cycle (lights out at 17:00 h). After familiarization in the Ann Arbor facility, rats were assigned to either AL or CR groups. At baseline (~ 14 weeks-old) prior to initiating the dietary protocol (CR group received 60–65% of AL intake daily for approximately 6 months as previously described [10]), the body mass of the groups were not significantly different (318.1 ± 3.3 g for AL and 315.7 ± 3.4 g for CR at baseline). When rats were 9 months of age, animals from both AL and CR groups were catheterized for blood collection as previously described [10]. Seven days after catheter placement, food was removed from the cages of all rats between 0700 and 0800 h, and body composition (body fat mass, lean mass, and free fluid) was measured in some of the rats using an NMR-based analyzer (Minspec LF90II, Bruker Optics; Billerica, MA). At 1200–1300 h, blood was collected from conscious rats and an aliquot was immediately used for glucose analysis by a glucometer (Accu-Chek Aviva, Roche, Indianapolis, IN). Additional blood was collected using heparinized capillary tubes (#22-362-566; Fisher Scientific, Hanover Park, IL). Blood was transferred to microcentrifuge tubes and centrifuged (1 min at 1500 g), with the resultant plasma fraction collected and stored at -80°C until analyzed.

ELISA kits (EMD Millipore, Billerica, MA) were used to measure plasma levels of insulin (#EZRMI-13K), adiponectin (#EZRADP-26K), BDNF (#CYT306), and FGF21 (#EZRMFGF21-26K). Multiplex bead kits (EMD Millipore) were used to measure C-peptide (#RMHMAG-84K), corticosterone (#RSH69K) and IL-1β (#RCYTOMAG-80K). Reagents and apparatus for SDS-PAGE and immunoblotting were from Bio-Rad Laboratories (Hercules, CA). Anti-FNDC5 antibody (#ab93373) was from Abcam (Cambridge, MA). Anti-myostatin antibody (#AB3239) was from EMD Millipore. Anti-rabbit IgG-horseradish peroxide conjugate (#7074) was from Cell Signaling Technology (Danvers, MA). Anti-myonectin antibody was a gift from Dr. G. William Wong at Johns Hopkins University School of Medicine. Myonectin and myostatin in plasma were determined by Western immunoblotting procedure as previously described [10]. Briefly, equal amounts of protein were separated by SDS-PAGE and transferred to nitrocellulose membranes. Equal loading was determined by the Memcode reversible protein stain kit for nitrocellulose membranes (PI-24580, Fisher). Plasma samples used for irisin analysis were prepared based on the procedures described by Bostrom et al. [15] including the removal of albumin/IgG (#122642; EMD Millipore) and deglycosylation using PNGase F (#P0704S; New England Biolabs, Ipswich, MA) prior to immunoblotting using the FNDC5 antibody. Immunoreactive proteins were quantified by densitometry (AlphaEase FC, Alpha Innotech, San Leandro, CA). Values are normalized to an average of the AL samples on the same blot. The HOMA-IR index [glucose (mg·dl^-1^) x insulin (μU·ml^-1^)/405] was calculated [25]. Unpaired Student’s t-tests were used for comparisons between AL and CR groups (SigmaPlot version 11.0; Systat Software, San Jose, CA). A P value ≤ 0.05 was accepted as statistically significant. Data are presented as mean ± SEM.

**Figure 1:**
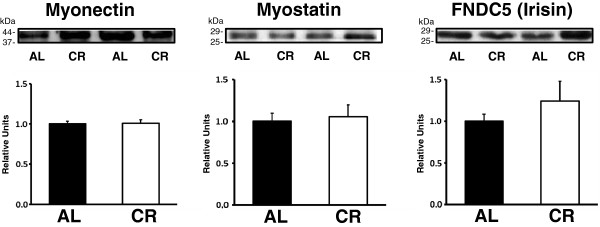
**Immunoblot analysis of plasma myonectin (42kDa), myostatin (26kDa), and FNDC5 (irisin; 26kDa).** Closed bars represent plasma from ad libitum-fed (AL) rats, and open bars represent plasma from calorie restricted (CR, 60-65% of AL daily intake) rats. Values are mean ± SEM. n=8-13 per treatment group.

CR rats compared to AL rats had significantly (P ≤ 0.05) lower body mass and absolute values (g) for fat mass, lean mass and free fluids (Table 1). When body composition results were expressed as relative values, CR rats versus AL rats had significantly (P ≤ 0.05) lower body fat percentage and higher lean body mass percentage with unaltered free fluid percentage. There were significant (P ≤ 0.05) decreases in plasma insulin and C-peptide levels in the CR compared to AL group (Table 2). There was no significant difference in glycemia for the AL and CR group (Table 2). The HOMA-IR index was significantly (P ≤ 0.05) lower for the CR versus AL group indicating improved insulin sensitivity in the CR rats (Table 2). There were significant (P ≤ 0.05) increases in plasma levels of both adiponectin and corticosterone in the CR versus AL group (Table 2). There were no significant diet-related effects on plasma levels of IL-1β, BDNF, FGF-21, myonectin, myostatin, or irisin (Table 2 and Figure 1).

**Table 1:** Body mass and composition

	**AL**	**CR**
**Body Mass (g)**	420.4 ± 19.3	263.5 ± 6.7*
**Fat Mass (g)**	65.2 ± 4.4	19.0 ± 1.9*
**Lean Mass (g)**	293.0 ± 10.3	205.5 ± 4.6*
**Free Fluid (g)**	31.9 ± 1.2	20.9 ± 0.5*
**Fat Mass (%)**	15.4 ± 0.5	7.1 ± 0.6*
**Lean Mass (%)**	70.0 ± 1.4	78.1 ± 0.4*
**Free Fluid (%)**	7.6 ± 0.2	7.9 ± 0.1

*Indicates a statistically significant difference (P ≤ 0.05) between the AL and CR groups as determined by unpaired Student’s *t*-test. Values are mean ± SEM for n=8-12 per treatment group. Body composition (fat, lean and free fluid masses) determined by nuclear magnetic resonance are expressed as both absolute (g, grams) and relative (%) values.

**Table 2:** Plasma glucose and protein concentrations and HOMA-IR index

	**AL**	**CR**
**Glucose (mg·dL**^**-1**^**)**	111.1 ± 2.3	105.9 ± 3.3
**Insulin (μU·mL**^**-1**^**)**	72.0 ± 10.1	29.5 ± 5.1*
**HOMA-IR Index**	19.3 ± 3.0	7.8 ± 1.4*
**C-peptide (pg·mL**^**-1**^**)**	847.3 ± 143.8	321.1 ± 58.2*
**Adiponectin (ng·mL**^**-1**^**)**	7275.3 ± 437.9	8723.4 ± 541.6*
**Corticosterone (ng·mL**^**-1**^**)**	113.3 ± 21.7	170.9 ± 26.1*
**IL-1β (pg·mL**^**-1**^**)**	20.8 ± 3.7	20.5 ± 5.9
**BDNF (pg·mL**^**-1**^**)**	89.1 ± 19.3	109.4 ± 22.6
**FGF21 (pg·mL**^**-1**^**)**	208.4 ± 32.6	160.4 ± 36.8

*Indicates a statistically significant difference (P ≤ 0.05) between the AL and CR groups as determined by unpaired Student’s *t*-test. Values are mean ± SEM for n=8-12 per treatment group. HOMA-IR Index; [glucose (mg·dl^-1^) x insulin (μU·ml^-1^)/405].

The primary goal of this study was to examine the influence of CR on the plasma concentrations of six proteins that were recently recognized to have metabolic functions (BDNF, FGF21, IL-1β, myonectin, myostatin and irisin) and that had not previously been studied in the context of CR. The results revealed no significant differences for any of these plasma proteins in AL compared to CR rats. It is notable that the CR protocol was accompanied by the expected changes in both body composition (lower percent fat mass and greater percent lean mass) and insulin sensitivity (improvement based on the reduction of the HOMA-IR index). The current results also confirmed earlier reports of CR-induced elevations in the plasma levels of adiponectin [1] and corticosterone [23]. Taken together, the data from this study indicate that alterations in plasma concentrations of BDNF, FGF21, IL-1β, myonectin, myostatin and irisin were not essential for the CR-related improvement in insulin sensitivity in rats. These novel results are valuable because they suggest that other mechanisms account for CR-induced improvement in insulin sensitivity.

It is important to recognize that the current results do not eliminate the possibility that one or more of these plasma proteins may be relevant for some of CR’s metabolic effects. It remains possible that CR may alter their diurnal fluctuations, that CR effects may be localized to protein concentrations in selected cells or tissues, or that CR may modify the sensitivity of target-cells. The current results also do not address the possibility for different outcomes during more brief or severe CR, in animals of other ages, in females, or in other species. Future studies should clarify possible CR effects on the originating tissues and time-courses for the secretion of these proteins and determine if the results for CR found in adult male rats are typical of other populations.

## Competing interests

The authors declare that they have no competing interests.

## Authors’ contributions

NS, CMC and GDC participated in the design and coordination of the study. NS and CMC performed the experiments and participated in the data acquisition. NS and GDC participated in the data analysis and drafting of the manuscript. All authors read and approved the final manuscript.
